# A Comparative Analysis of Long‐Term Effective Population Sizes Across Eukaryotes

**DOI:** 10.1111/mec.70265

**Published:** 2026-02-12

**Authors:** Loveday Lewin, Adam Eyre‐Walker

**Affiliations:** ^1^ School of Life Sciences University of Sussex Brighton UK

**Keywords:** comparative population genomics, effective population size, genetic drift, mutation rate, nucleotide diversity

## Abstract

The effective population size (*N*_e_) is a fundamental parameter in population genetics. Despite its central importance, there are relatively few estimates of *N*_e_ available and there have been limited attempts to compare values across eukaryotes. Here, we estimate long‐term effective population sizes for 120 species, broadly distributed across the eukaryotic tree of life, using nucleotide diversity and direct mutation rate estimates. We find that *N*_e_ varies by nearly 4 orders of magnitude and that it shows strong phylogenetic structure across broad taxonomic scales but not within individual lineages. Phylogenetically controlled regressions reveal that *Nₑ* correlates with key life history traits, including generation time and propagule size, and that nucleotide diversity serves as a useful proxy for *Nₑ*. Finally, we show that small *Nₑ* is generally associated with a reduction in the efficacy of natural selection, as indicated by an elevated ratio of non‐synonymous to synonymous diversity (π_N_/π_S_), but not with an increase in genome size after accounting for phylogenetic non‐independence. These results provide a broad comparative perspective on the factors driving variation in *Nₑ* and its evolutionary consequences across eukaryotes.

## 1 Introduction

Random genetic drift is one of the most important population genetic processes, impacting the levels of neutral and nearly neutral genetic diversity that can be maintained in a population, the efficacy of natural selection, patterns of linkage disequilibrium and levels of population differentiation in subdivided populations (Charlesworth 2009). The power of drift is inversely proportional to the effective population size (*N*_e_), such that drift is strong in species or populations with small *N*_e_ but weak in species or populations with large *N*_e_. However, despite its central role in evolutionary biology, there have been few attempts to estimate long‐term *N*_e_ across a broad range of species and view these estimates in a comparative framework.

Multiple definitions of *Nₑ* exist, each reflecting different evolutionary processes and temporal scales (Wang 2005; Wang et al. 2016; Nadachowska‐Brzyska et al. 2022). Several methods, such as tracking changes in allele frequencies over a small number of generations, aim to estimate contemporary *N*_e_, usually for a single, localised population (Luikart et al. 2010). These estimates are generally low: a meta‐analysis reported a median contemporary *N*_e_ of just 260 in eukaryotes (Palstra and Ruzzante 2008). Such contemporary estimates are valuable in conservation and population management contexts (Clarke et al. 2024), but will not necessarily be relevant to understanding the long‐term evolutionary consequences of drift on levels of genetic variation and the efficiency of selection. For this, we need to estimate *N*_e_ over the age of the genetic variation in the population, which is most simply obtained by dividing the present‐day nucleotide diversity by the mutation rate, since the level of neutral diversity is expected to be 4*N*_e_μ, where μ is the mutation rate (Watterson 1975). Our estimates of *N*_e_ can be viewed as the population size of randomly mating individuals in a stationary population that would give the same diversity, but it is also an estimate of the average rate of coalescence (Charlesworth 2009). We refer to these as long‐term estimates, though they are also known as estimates of the coalescent *N*_e_ (Sjödin et al. 2005; Wakeley and Sargsyan 2009).

A number of such estimates have been made, and it is clear that they vary considerably between species, from organisms such as ourselves with an *N*_e_ of approximately 20,000 to bacteria such as *Vibrio cholerae* with an *N*_e_ of over 400 million (Lynch et al. 2016). Generally, these estimates of *N*_e_ have been made to test whether some other factor, such as the mutation rate (Bergeron et al. 2023; Lynch et al. 2023), transposable element content (Marino et al. 2024), or rate of adaptive evolution (Gossmann et al. 2012), is correlated to *N*_e_, with limited exploration of the *N*_e_ estimates themselves. Here, we rectify this by making *N*_e_ the focus of our investigation.

Quantifying the variation in *N*_e_ across eukaryotes is important for several reasons. First, establishing typical *N*_e_ values within major phylogenetic groups can provide insights into how the power of drift differs across evolutionary lineages. Second, examining how *N*_e_ varies across a broad phylogeny can reveal the extent to which *N*_e_ as a trait is constrained by evolutionary history versus being flexibly shaped by species traits and ecology. Third, identifying systematic correlates of *N*_e_, such as demographic or life history traits, can help uncover the drivers of its variation. Additionally, a comprehensive dataset of *N*_e_ estimates provides an opportunity to assess the performance of widely used proxies of *N*_e_ such as nucleotide diversity and the ratio of non‐synonymous to synonymous diversity (π_N_/π_S_). Finally, *N*_e_ is predicted to affect key genomic features such as genome size (Lynch and Conery 2003), and comparative data provides an opportunity to evaluate this expectation across broad evolutionary scales.

Over the last decade, the revolution in high‐throughput sequencing technologies has resulted in a dramatic increase in the availability of experimental mutation rate estimates from pedigrees and mutation accumulation lines (Wang and Obbard 2023). By combining this mutation rate data with nucleotide diversity estimates, we are now able to generate robust long‐term *N*_e_ estimates for 120 diverse eukaryotic species. We leverage this dataset to address the key questions detailed above.

## 2 Materials and Methods

### 2.1 Estimating *N*_e_ Across Eukaryotes

To estimate effective population sizes (*N_e_*) across a wide range of species, we collected data on mutation rates and nucleotide diversity from the literature. De novo mutation rate estimates were primarily obtained from a meta‐analysis by Wang and Obbard (2023). An additional literature search was performed following their search strategy for the period from 22 September 2022 to 15 April 2024 to ensure the inclusion of more recently published data, yielding an additional 30 estimates and bringing the total number of species with genome‐wide mutation rate estimates to 156 (Table S2). All estimates were based on sequencing of closely related individuals in pedigrees or mutation accumulation lines. Where original studies provided 95% confidence intervals, these were recorded. For cases where uncertainty measures were unavailable, binomial confidence intervals were estimated using the *binconf()* function from the Hmisc R package (Harrell 2024) based on the number of mutation events and callable sites. For species with multiple mutation rate estimates from different studies, we calculated a weighted mean using the number of trios in parent‐offspring studies or the product of the number of lines and generations in mutation accumulation studies as weights. 95% confidence intervals for the weighted means were calculated by propagating the within‐study variances of individual estimates, such that the final uncertainty reflects the cumulative sampling error across studies.

Corresponding nucleotide diversity estimates were sourced from a variety of existing compilations (Leffler et al. 2012; Romiguier et al. 2014; Corbett‐Detig et al. 2015; Chen et al. 2017; Buffalo 2021) and the wider literature (see Table S3). We chose to use nucleotide diversity (π) as our diversity metric because it is related to the average rate of coalescence, it is widely reported, and also because it is expected to be fairly robust to variation in sample sizes and sampling strategies across species (Subramanian 2016; Aoki et al. 2023). An alternative would be to use Watterson's (1975) estimator of 4*N*_e_μ, θ_w_, but this has no clear interpretation once the assumptions of the model used to derive the estimator are violated. We found that, across 20 species for which both metrics were reported, there was a significant correlation between π and θ_w_ (Spearman's *ρ* = 0.93, *p* < 0.001) and no significant difference between them (*p* = 0.68).

We assembled nucleotide diversity estimates for 139 species; these include both genome‐wide and synonymous site diversity estimates. We find no significant difference between genome‐wide and synonymous site estimates in those species for which we have both (22 species; geometric means of 0.0066 (genome‐wide) and 0.0079 (synonymous); *t*‐test on log‐transformed values, *t* = −1.85, df = 21, *p*‐value = 0.079), so we treated the two types of estimate as equivalent. For studies that also provided estimates of non‐synonymous diversity, these were recorded to include in analysis of the relationship between *N*_e_ and π_N_/π_S_. Diversity estimates came from a range of sampling strategies, from single geographically defined populations to broader sampling across the species range. For species with diversity estimates from multiple populations, we averaged the values rather than using a pooled estimate across populations, as intra‐population diversity better reflects *N_e_* in subdivided species (Charlesworth and Charlesworth 2010).

Eight species that are primarily domesticated or livestock animals were removed from the dataset because their genetic diversity estimates are likely not representative of natural populations due to extensive human intervention. For each of the remaining 131 species in our dataset with both a mutation rate and a diversity estimate, *N*_e_ was estimated as:

$$N_{e}=\frac{\pi }{2\mathrm{x\mu}}$$

where π is nucleotide diversity, that is, the average number of pairwise nucleotide differences over all sampled pairs, x is the ploidy of the organism and μ is the mutation rate per site per generation (see Table S1 for *N*_e_ estimates). We will refer to these estimates as *N̂*_e_. CIs were inferred by propagating the error from the mutation rate estimates onto the *N̂*_e_ estimates, as we expect these to be a critical source of uncertainty associated with the estimation of long‐term *N*_e_ (Waples 2010).

### 2.2 Life History, Demographic and Genomic Trait Data

Species‐specific data on life history traits (adult body mass, generation time, maximum longevity, propagule size) were collected from the literature (see Table S4 for details). Romiguier et al. (2014) showed that nucleotide diversity is strongly correlated to propagule size, which they define as the maximum linear measurement of the individual when it leaves parental care; for example, for species that provide no parental care this is the maximum linear dimension of the egg. For mammals lacking existing estimates of propagule size, this was approximated following the relationship described in Romiguier et al. (2014):

$$\text{Propagule size}=\text{adult body length}\times {\left(\frac{\text{body mass}\;\mathrm{at}\;\text{weaning}}{\text{adult body mass}}\right)}^{3}$$

Data were also collected on species population density and geographic ranges (Table S5). If an appropriate range estimate was not available, we produced estimates based on occurrence records from the Global Biodiversity Information Facility (GBIF) with the gbif.range R package (Chauvier et al. 2022), which generates species maps using georeferenced observations and ecoregion polygons. We produced range maps for 42 species and inspected the resulting plots for consistency with known range information. In a number of cases, observations from specific countries were manually removed to exclude occurrences outside the species' known native range, based on range descriptions from the IUCN or published literature (Table S6).

Data on genome sizes (*C*‐values) were obtained for 63 animal and plant species from the Animal Genome Size Database (Gregory 2025) and Plant DNA *C*‐value Database (Leitch 2019), respectively.

### 2.3 Statistical Analyses

We investigated the relationships between *N̂*_e_, mutation rates, genetic diversity and various species‐specific traits using phylogenetic generalised least squares (PGLS) regressions (Freckleton et al. 2002). This method accounts for the non‐independence of trait values due to shared evolutionary history by incorporating a phylogenetic covariance matrix into the regression model.

For the full dataset of 131 species with *N*_e_ estimates, a phylogeny was produced using timetree.org (Kumar et al. 2022), which was then used to conduct PGLS analyses with the R package caper (Orme et al. 2023). The full phylogeny was pruned for each individual model based on the species that had complete data for the trait in question. 11 species were lost from our analysis since Timetree had no data on them or a closely related species; this meant that the following analyses were ultimately performed on a final set of 120 species. Because of the diverse range of species included in the dataset, all analyses were performed on log transformed variables as values varied over orders of magnitude.

To test for heterogeneity in *N̂*_e_ within each taxonomic group, we performed a weighted chi‐squared test for variance, also known as Cochran's *Q* test in meta‐analyses. Log‐transformed *N̂*_e_ and their variances were computed from reported 95% confidence intervals. Within each group, a weighted mean was calculated using inverse‐variance weighting, and a chi‐squared statistic was computed to test for excess variability.

## 3 Results

### 3.1 Variation in Effective Population Sizes Across Eukaryotes

**FIGURE 1:**
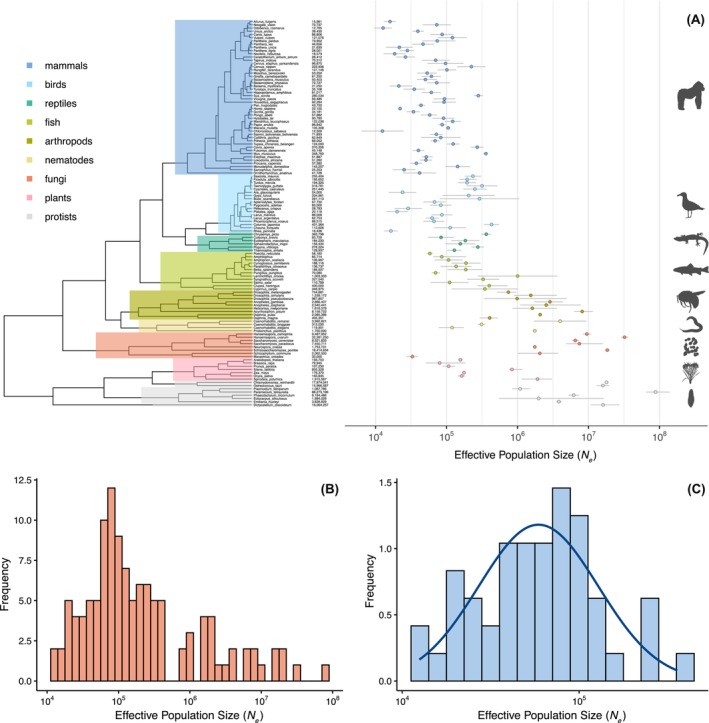
*Variation in N̂*_e_ *across the eukaryotic phylogeny*. (A) An overview of estimated effective population sizes (*N̂*_e_) in eukaryotes (all estimates can also be found in Table S1). Left: Phylogenetic tree generated using timetree.org and plotted using ggtree in R (Yu et al. 2017) with major taxonomic groups colour‐coded according to the legend. Raw *N*_e_ estimates are provided next to species names. Right: Species‐specific *N*_e_ estimates plotted on a logarithmic scale with error bars indicating 95% confidence intervals. Silhouettes sourced from PhyloPic (https://www.phylopic.org/permalinks/a9bea0f564201fa94c92bf238e471a0b6f8b7e0a13d755e4b15842c879f70e44). (B) Histogram of *N̂*_e_ estimates across all species plotted on a logarithmic scale. (C) Histogram of *N̂*_e_ estimates across mammals plotted on a logarithmic scale fitted with a log‐normal distribution.

We have calculated long‐term estimates of effective population size for 120 species broadly distributed across the eukaryotic phylogeny using nucleotide diversity and experimental mutation rate estimates sourced from the literature. We found substantial variation in *N̂*_e_ across eukaryotes, spanning almost four orders of magnitude (Figure 1). The highest *N̂*_e_ was for the single‐celled protist *Paramecium tetraurelia* (88.3 million), while the lowest *N̂*_e_ was found in the green monkey, *Chlorocebus sabaeus* (12,500). On average, protists and fungi had the largest median *N̂*_e_, with 16 million and 8.5 million, respectively. In contrast, mammals had the smallest median *N̂*_e_ (61,000), followed by birds (99,000).

Across the entire phylogeny, we identified a strong phylogenetic signal for *N̂*_e_ (Pagel's *λ* = 0.95, *p* < 0.001, Table 1), suggesting that related species tend to have similar effective population sizes due to their shared evolutionary history. However, this phylogenetic effect appears to be primarily driven by broad‐scale evolutionary trends, as analyses within individual taxonomic groups revealed little to no evidence of significant phylogenetic signal (Table 1). The only exceptions are within birds, where variation in *N̂*_e_ between passerine and non‐passerine species may be driving the observed signal, and fungi, where we have very few species and substantial variation. Note that unless otherwise stated, all analyses were performed on log values of all variables, although for simplicity we refer to them by the name of the factor—for example, all analyses use log(*N̂*_e_), which we refer to as *N̂*_e_.

**TABLE 1:** Estimates of phylogenetic signal (Pagel's *λ*) for estimated effective population size (*N̂*_e_) within taxonomic groups. *λ* values closer to 1 indicate strong phylogenetic dependence while values closer to 0 suggest little to no phylogenetic structure.

Group	Species (*n*)	Pagel's *λ*	Pagel's *λ* *p*	Cochran's *Q p*
Mammals	48	0.00	1.0	< 0.001
Birds	18	1.02	< 0.001	< 0.001
Reptiles	6	0.00	1.0	< 0.001
Fish	12	0.00	1.0	< 0.001
Arthropods	9	0.97	0.59	< 0.001
Fungi	8	1.10	< 0.05	< 0.001
Plants	7	1.03	0.39	< 0.001
Protists	8	0.00	1.0	< 0.001
All	120	0.95	< 0.001	

*Note:* The number of species included in the analysis for each group (*n*) is provided. Also given is the result of a Cochran's *Q* test for heterogeneity in log(*N̂*_e_) values.

The presence of phylogenetic signal demonstrates that *N̂*_e_ varies significantly between species at large scales (e.g., protists have substantially larger *N*_e_ estimates than mammals). However, even within groups lacking a detectable phylogenetic signal, we observe significant variation in *N̂*_e_ using Cochran's *Q* test (Table 1). These results suggest that factors beyond shared evolutionary history, such as life history traits, ecological dynamics and population structure, play a role in shaping variation in *N̂*_e_ at finer taxonomic levels.

In mammals, we have the opportunity to estimate the distribution of *N̂*_e_ across species because we have significant variation in *N̂*_e_, a substantial number of estimates and no phylogenetic signal, so sampling of species should not matter so long as it is random with respect to *N̂*_e_. We find that *N̂*_e_ varies from several thousand to several hundreds of thousands of individuals, with most mammalian species having *N*_e_ estimates in the tens of thousands (Figure 1C). Our analysis suggests that there are unlikely to be many mammals with *N*_e_ estimates of less than 10,000 or more than 1 million.

### 3.2 Biological Correlates of *N*_e_

A number of biological factors have been shown to correlate to nucleotide diversity, including estimates of census population size (reviewed by (James and Eyre‐Walker 2020; Buffalo 2021)), propagule size (Romiguier et al. 2014), longevity (Leffler et al. 2012) and body size (Mackintosh et al. 2019). However, in each case, it remains unclear whether the correlation arises because the trait influences the mutation rate, *N*_e_ or both. It is therefore valuable to directly characterise the relationships between these traits and *N*_e_ to disentangle their relative contributions. Furthermore, identifying strong biological correlates of *N*_e_ that are easy to measure and comparable across species could provide a practical means of predicting *N*_e_ in species lacking extensive sequencing or mutation rate data. To explore this, we compiled data on key demographic and life history traits for many of the species in our dataset, including range size, population density, adult body mass, longevity, generation time and propagule size.

Census population sizes (*N*_c_) are notoriously difficult to estimate due to the logistical challenges of surveying natural populations, and such estimates are generally associated with very large margins of error. We therefore attempted to estimate the census population size using range size and population density estimates, the product of which can be considered an approximation for *N*_c_ (Buffalo 2021). We note that our approximations of *N*_c_ are based on reported population densities that do not always distinguish mature adults from juveniles. As a result, these *N*_c_ values should be interpreted as broad estimates rather than precise counts of mature individuals, which would be the gold standard for a comparative study of this nature (Frankham 2007).

We find that *N̂*_e_ is significantly positively correlated to population density and to the product of range size and population densities but not to range size alone (Table 2; note that all regression analyses control for phylogeny using PGLS). Population density is also a challenging metric to estimate, but can be approximated based on the established macroecological relationship between density and body size (Damuth 1987). We find that *N̂*_e_ is significantly positively correlated to range size divided by body mass—our proxy for population density. Together these results suggest, as expected, that *N̂*_e_ is positively correlated to *N*_c_. However, the slope of this relationship is very shallow (PGLS slope = 0.09, Table 2), such that increasing *N*_c_ by 10‐fold only increases *N̂*_e_ by 1.23‐fold.

**TABLE 2:** Summary of PGLS regressions of effective population size estimates (*N̂*_e_) and nucleotide diversity (π) on a range of demographic and life history traits.

Focal demographic trait	Species (*n*)	PGLS slope (*N*_e_)	Adj. *R* ^2^ (*N*_e_)	PGLS slope (π)	Adj. *R* ^2^ (π)
Geographic range (km^2^)	73	0.09	0.01	0.02	−0.01
Population density (*n* per km^2^)	59	0.11**	0.13	0.08**	0.11
Range/body mass	65	0.12***	0.16	0.07*	0.12
Range × density (*N*_c_ proxy)	50	0.09**	0.12	0.06**	0.12

Focal life history trait	Species (*n*)	PGLS slope (*N*_e_)	Adj. *R* ^2^ (*N*_e_)	PGLS slope (π)	Adj. *R* ^2^ (π)
Adult body mass (g)	82	−0.11***	0.13	−0.06**	0.07
Generation time (year)	120	−0.51***	0.47	−0.17**	0.05
Maximum longevity (year)	82	−0.43***	0.15	−0.26*	0.07
Propagule size (cm)	55	−0.29**	0.17	−0.11	0.02

Abbreviation: PGLS, phylogenetic generalised least squares.

*** *p* < 0.001.

** *p* < 0.01.

* *p* < 0.05.

**FIGURE 2:**
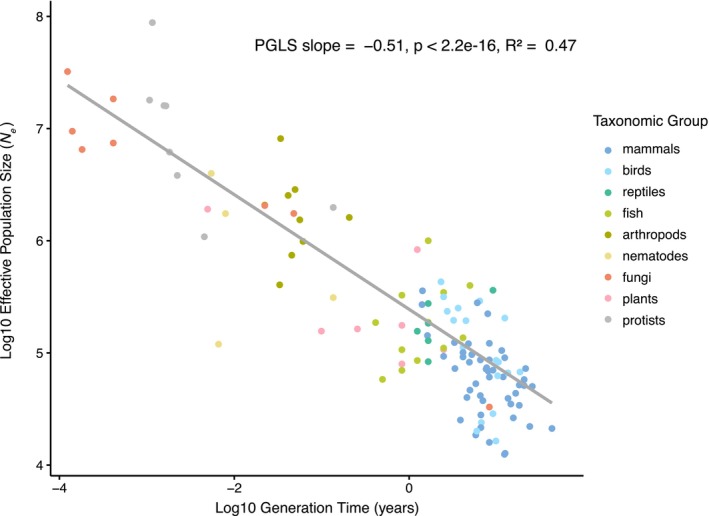
PGLS regression of effective population size estimates (*N̂*_e_) on generation time on a log–log scale. Species are coloured based on their taxonomic group.

We identified several other traits that show significant correlations with *N̂*_e_ (Table 2). Among these, generation time exhibited the strongest relationship, with a significant negative slope (*p* < 0.001, *R* ^2^ = 0.47, Figure 2) indicating that species with longer generation times tend to have lower effective population sizes. Additionally, adult body mass, propagule size and longevity were all significantly negatively correlated with *N̂*_e_, suggesting that larger, longer lived species with greater parental investment tend also to have smaller effective population sizes (Table 2).

Notably, the vast majority of traits showed stronger correlations with *N̂*_e_ than with nucleotide diversity (Table 2), even though our estimates of *N̂*_e_ have a larger sampling error than the original π estimates (the error variance associated with *N̂*_e_ is a product of both the error in π and the error in the mutation rate estimate, with the latter expected to be relatively large). This likely reflects the fact that *N̂*_e_ provides a less noisy measure of how life history traits influence population dynamics, as it isolates the effects of genetic drift without the added variability in mutation rates. In contrast, π is jointly determined by both *N̂*_e_ and the mutation rate, meaning that variation in μ across species could obscure the underlying relationships between life history traits and genetic diversity.

Many of these traits are correlated among themselves (Figure S1). In an attempt to determine which factors are the most important in explaining variation in *N̂*_e_, we performed a series of phylogenetically controlled multiple regression analyses prioritising the factors with the highest *R* ^2^ values. Due to gaps in data availability, each model included only two to three traits to maximise statistical power. These models were then compared to identify those with the greatest explanatory power (see Table S7).

Among the two‐factor models, generation time, propagule size and a proxy for population density (range size divided by adult body mass) frequently emerged as the dominant predictors, exhibiting the highest standardised slopes. We take these to be the traits with the greatest predictive power for *N̂*_e_; a model including all three yielded the highest adjusted *R* ^2^ of 0.64.

### 3.3 *N*_e_ and Genetic Diversity

Genetic diversity is frequently used as a proxy for effective population size in studies where *N*_e_ is not estimated directly. However, levels of neutral genetic diversity are expected to depend upon both *N*_e_ and the mutation rate. By considering the extent to which each of these components can explain variation in diversity, we can evaluate the validity of using diversity as a proxy for *N*_e_.

**FIGURE 3:**
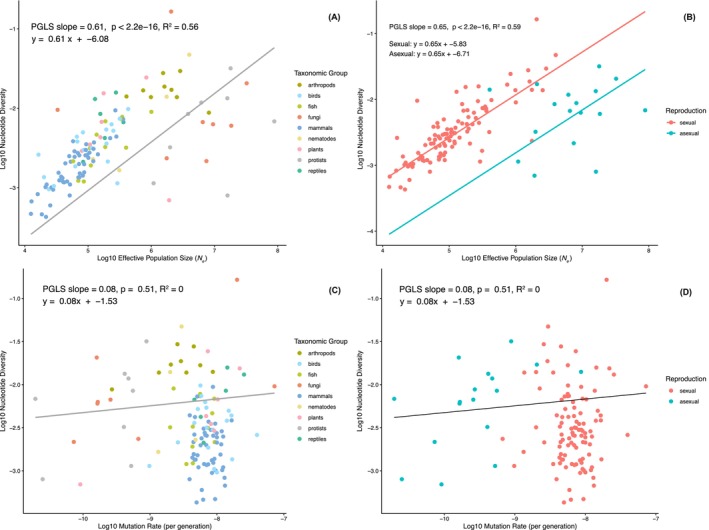
(A) PGLS regression of nucleotide diversity (π) on effective population size estimates (*N̂*_e_) on a log–log scale. Species are coloured based on their phylogenetic group. (B) PGLS regression of nucleotide diversity (π) on effective population size estimates (*N̂*_e_) on a log–log scale with reproductive strategy as a fixed effect. Species are coloured based on their reproductive strategy. (C) Phylogenetic generalised least squares (PGLS) regression of nucleotide diversity (π) on mutation rate (μ) on a log–log scale. Species are coloured based on their phylogenetic group. (D) Phylogenetic generalised least squares (PGLS) regression of nucleotide diversity (π) on mutation rate (μ) on a log–log scale. Species are coloured based on their reproductive strategy.

Using a PGLS regression, we identified a strong positive correlation between nucleotide diversity and *N̂*_e_ (*R* ^2^ = 0.56, Figure 3A). We do not give a *p*‐value associated with this correlation because our estimates of *N*_e_ depend on the estimates of nucleotide diversity; hence, sampling error in our estimate of nucleotide diversity could generate an artefactual correlation between *N̂*_e_ and π. To address this, we made use of the fact that for some species we have multiple independent estimates of nucleotide diversity. We split these estimates into two separate datasets and used one to calculate *N̂*_e_ and the other as our estimate of diversity (and vice versa; Figure S2A,B). The correlation remained significant and similar to that in our original analysis (Figure S2A: *R* ^2^ = 0.57, *p* = 8.4e‐06; Figure S2B: *R* ^2^ = 0.54, *p* = 3.2e‐05), reflecting both the fact that there is a genuine correlation between *N̂*_e_ and nucleotide diversity, and the fact that there is limited sampling error in our estimates of nucleotide diversity.

Qualitatively, some fungi and protist species appeared to deviate from the relationship between *N̂*_e_ and π. Given that alternative reproductive strategies are expected to have a significant impact on key population genetic parameters, including *N*_e_ and mutation rates (Glémin and Galtier 2012), one possible explanation is that the asexual mating systems employed by many of these species are influencing the scaling of this relationship. Consistent with this, we find that reproductive mode is significant (*p* < 0.001) if it is introduced as a fixed effect into the PGLS model, and the fit of the model improves slightly (*R* ^2^ = 0.59). However, we find no evidence of an interaction between reproductive mode and the slope. Overall, this implies that for a given *N̂*_e_, asexual species exhibit significantly lower nucleotide diversity than sexual species, even though the overall relationship between diversity and *N̂*_e_ remained consistent across reproductive strategies.

In contrast, we found no significant relationship between nucleotide diversity and the mutation rate per generation (*p* = 0.51, *R* ^2^ = 0, Figure 3C,D), irrespective of phylogenetic group or reproductive strategy. These results indicate that most of the ~700‐fold variation in nucleotide diversity observed across these species can be attributed to variation in *N̂*_e_, rather than variation in the mutation rate.

### 3.4 *N*_e_ and the Efficiency of Natural Selection

According to the nearly neutral theory of molecular evolution, the effectiveness of natural selection in removing weakly deleterious mutations is expected to depend on effective population size (Charlesworth 2009). As *N*_e_ decreases, the efficacy of selection decreases as genetic drift becomes more influential and increasingly dictates the fate of effectively neutral mutations. The ratio of nonsynonymous to synonymous nucleotide diversity (π_N_/π_S_) is a commonly used measure of the efficiency of natural selection, as it quantifies the proportion of nonsynonymous mutations that are effectively neutral. Under nearly neutral theory, we expect this proportion of effectively neutral mutations to be a function of *N*_e_—to the extent that it is frequently used as a proxy for *N*_e_—with smaller *N*_e_ leading to a higher proportion of effectively neutral mutations due to the increasing influence of genetic drift.

**FIGURE 4:**
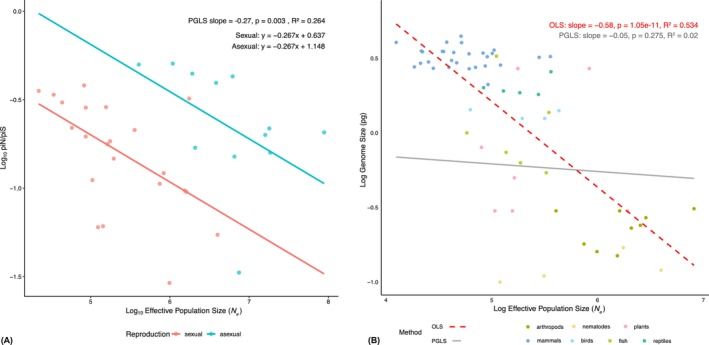
(A) PGLS regression of the ratio non‐synonymous to synonymous diversity (π_N_/π_S_) on estimated effective population size (*N̂*_e_) on a log–log scale with reproductive strategy included as a fixed effect. Species are coloured based on their reproductive strategy. (B) Comparison between a PGLS regression (grey line) and an OLS regression (red dotted line) of genome size on effective population size (*N̂*_e_) on a log–log scale. Species are coloured based on their taxonomic group.

When examining all species together, we detected no significant relationship between *N̂*_e_ and π_N_/π_S_ (*p* = 0.22, *R* ^2^ = 0.02, Figure S3). However, the relationship becomes significantly negative if we introduce reproductive mode as a fixed effect (*p* = 0.003, *R* ^2^ = 0.26, Figure 4A). This revealed that asexual species have significantly higher π_N_/π_S_ ratios than sexual species (reproductive mode effect *p*‐value = 0.002), indicating systematically weaker purifying selection in asexual lineages. However, we find no evidence of an interaction between *N̂*_e_ and reproductive mode. Hence, we have evidence that π_N_/π_S_ decreases with increasing *N̂*_e_ and that the intercept of this relationship differs between sexuals and asexuals, with asexuals showing less effective selection for a given *N̂*_e_.

The decreased efficiency of natural selection in populations with small *N*_e_ has also been predicted to produce a negative association between *N*_e_ and genome size, with small populations expected to be less effective at removing slightly deleterious mutations that increase genome size (Lynch and Conery 2003; Lynch 2007). We tested this hypothesis using a commonly employed measure of genome size: *C*‐values (the amount of DNA in a haploid nucleus). As expected, a simple OLS regression revealed a significant negative relationship between *N̂*_e_ and genome size (*p* = 1.05e−11, *R* ^2^ = 0.53, Figure 4B). However, this relationship became nonsignificant when phylogenetic history was accounted for using PGLS (*p* = 0.28, *R* ^2^ = 0.02, Figure 4B), indicating that the apparent association may be driven by phylogenetic structure rather than a direct causal link between *N̂*_e_ and genome size.

## 4 Discussion

We have performed a comparative analysis of long‐term effective population size estimates produced from nucleotide diversity and experimentally derived mutation rate data. In a trivial sense, our estimates of *N̂*_e_ are simply an estimate of the size of an idealised, stationary population of randomly mating individuals that would produce the observed level of diversity given the mutation rate. However, more fundamentally, this is an estimate of (half) the average time to coalescence, measured in generations. As such, it encompasses the effects of the many factors that shape genealogies including changes in population size, breeding systems and nonequilibrium dynamics. We find that *N̂*_e_ varies substantially between species from the thousands to the tens of millions. This is roughly an order of magnitude more than that reported by Galtier and Rousselle (2020), who estimated that the strength of genetic drift varies by three orders of magnitude between primates and fruit flies based on deleterious mutation load. Our broader phylogenetic sampling, which includes plants and unicellular eukaryotes in addition to animals, likely contributes to this increased variation. We find that *N̂*_e_ shows phylogenetic inertia over broad, but not fine, taxonomic scales. Among the strongest correlates of long‐term *N̂*_e_ are generation time, propagule size and geographic range scaled by body mass—a proxy for population density. Nucleotide diversity also shows a strong positive relationship with *N̂*_e_, and we show that this is unlikely to be an artefact of correlated sampling errors between the two parameters. Finally, we explore the expected consequences of variation in *N̂*_e_, showing that species with smaller *N̂*_e_ tend to have higher ratios of nonsynonymous to synonymous nucleotide diversity, consistent with less efficient selection in species with small *N̂*_e_. However, we find no evidence that smaller *N̂*_e_ is associated with increased genome size.

### 4.1 Limitations

Estimates of long‐term *N*_e_ based on the relationship π = 4*N*_e_μ are inherently limited by the accuracy of the two component variables: nucleotide diversity (π) and mutation rates (μ). Uncertainties or biases in the estimation of either one will propagate directly onto the final *N*_e_ estimate, potentially leading to significant deviations from the true long‐term effective population size.

While direct mutation rate estimation methods represent a substantial advance over earlier approaches based on long‐term substitution rates, they are still subject to significant uncertainty. The rarity of de novo mutations means that pedigree‐based mutation rate estimates are highly sensitive to methodological differences, including variability in sequencing coverage, the filtering of candidate DNMs, and both the quality and choice of reference genome. It has been shown that estimates for the same parent‐offspring trio can differ by as much as twofold, depending solely on the analysis pipeline employed (Bergeron et al. 2022). The magnitude of this disparity could produce similarly divergent *N*_e_ estimates.

To account for this uncertainty in the available mutation rate estimates, we propagated the confidence intervals associated with each mutation rate onto the resulting *N̂*_e_ estimates (Figure 1A). These intervals were based on the number of detected de novo mutations and the total number of callable sites in the sequenced genomes, reflecting the statistical confidence around the observed mutation count. However, it is important to note that these intervals may underestimate the true uncertainty by excluding additional, true biological variability in mutation rates between families that is not captured from restricted pedigree estimates. This issue is mitigated to an extent in our dataset because the mutation rates for most species are averaged from multiple independent studies, or at the very least from multiple trios or mutation accumulation lines, meaning they should be capturing some of this between‐family variance.

Unlike this simple underestimation of variance, a more concerning potential source of systematic bias in the mutation rate data is differences between the conditions under which mutation rates are estimated and those experienced by populations in the wild. In particular, discrepancies in generation time between laboratory conditions and natural settings could skew mutation rate estimates that are expressed on a per generation scale (Bergeron et al. 2023). This is likely to be a more significant issue in longer lived species where experimental generation times are often artificially shortened, potentially leading to systematic underestimation of μ and, consequently, overestimation of *N*_e_. These considerations highlight the fact that, while our *N̂*_e_ estimates provide a valuable, comparative view of drift across eukaryotes, they should be interpreted with some caution. Refining these analyses will depend on future work to improve the accuracy, precision and biological relevance of direct mutation rate estimates.

While the basic sampling error associated with estimating nucleotide diversity from sequence data is generally expected to be lower than that for mutation rates, thanks to large genomic datasets and to recombination creating many independent genealogies within a single genome, there is significant potential to introduce bias into long‐term *N*_e_ estimates through the spatial sampling strategy of sequenced individuals. Estimates of *N*_e_ derived from π reflect an average across the sampled populations. The presence of population structure, where a species is divided into genetically distinct subpopulations with limited gene flow, can significantly influence measures of π and, consequently, the interpretation of *N̂*_e_. Because our study relied on obtaining diversity estimates for a wide variety of species using publicly available data, it was not always possible to account for the spatial scale and structure of the available estimates. The most significant potential problem arises if these estimates were based on *ad hoc* sampling across a species' range without assessment of population structure. In these cases, if there was structure present, then pooling individuals together and calculating a single diversity estimate would inflate π relative to the average diversity within subpopulations, which is theoretically the most appropriate measure for comparing *N*_e_ across species (Charlesworth 2009).

However, we have a number of reasons to expect that this potential issue would not substantially affect our main conclusions. First, meta‐analyses have suggested that *F*_ST_, a widely used measure of between‐population differentiation, is generally low across many of taxa, indicating limited genetic structure and reasonably high levels of gene flow (Morjan and Rieseberg 2004). Even small amounts of migration between demes over long timescales are expected to normalise genetic diversity towards the global, species‐wide diversity, regardless of initial sampling strategy under models with conservative migration (a population receives as many migrants as it loses) (Waples 2010; Hare et al. 2011), although the relationship between global and local diversity is more complex under models of non‐conservative migration (Whitlock and Barton 1997). To assess the robustness of our findings to population structure, we replicated the key analyses of the paper on a subset of species for which we were relatively confident that there was limited population structure in the sampling of diversity estimates. This subset included species with sampling from a single, well‐defined population and species for which previous analysis indicated high levels of gene flow (*F*_ST_ < 0.2) within the sampled range. We found that the results from this subset analysis did not materially differ from those performed on the full dataset (see Figure S4). This suggests that, while acknowledging the inherent limitations of our data collection approach, the potential impacts of population structure are unlikely to invalidate the key findings of our work regarding long‐term *N*_e_.

Besides potential population structure, several additional sources of bias may affect the estimates of nucleotide diversity used in this study. Estimates of π are sensitive to a number of methodological choices: for example, mapping reads to a heterospecific reference genome (Akopyan et al. 2025) or failing to account for missing data when calculating π from VCF files can both result in downwardly biassed estimates. Given our reliance on existing published data, we were unable to correct for these issues directly. Nevertheless, future best practices for population genetic analyses of this kind should include the use of either VCFs that report both variant and invariant sites or software that explicitly accounts for missing data, such as pixy (Korunes and Samuk 2021), as well as alignment to high‐quality, conspecific reference genomes. In the longer term, reference‐free pangenomic approaches may help to eliminate reference genome bias altogether (Lin et al. 2024; Ahmad et al. 2025).

Finally, we assume that our diversity estimates are derived from neutral parts of the genome. In reality, however, certain genomic regions will be subject to selection and therefore have fewer SNPs. Given that the fraction of the genome under selection is unknown for most species, our estimates of neutral diversity and *N̂*_e_ are likely to be underestimates. It has been estimated that the fraction of the genome subject to selection is approximately 8% in humans (Rands et al. 2014), but may be as high as 70% in *Drosophila melanogaster* (Andolfatto 2005). Nevertheless, much of this selection is thought to be weak and thus unlikely to substantially impact overall levels of diversity.

### 4.2 Diversity, Mutation and *N*_e_

According to population genetic theory, levels of neutral genetic diversity are determined by the product of *N*_e_ and the mutation rate (Watterson 1975). In this study, we aimed to assess the extent to which each of these elements contributes to shaping variation in nucleotide diversity across species. Given the relatively limited variation in mutation rates observed among eukaryotes, especially within phylogenetic groups, it is perhaps unsurprising that we find mutation rate explains little, if any, of the observed variation in nucleotide diversity (Figure 3C). While correlations between diversity and mutation rate have been reported within genomes, that is, across different genomic regions (Castellano et al. 2020), such relationships are not necessarily expected to translate across species—these being fundamentally different levels of inquiry.

Instead, our results suggest that much of the interspecific variation in nucleotide diversity is driven by variation in *N̂*_e_, as indicated by the strong correlation between the two in Figure 3A. However, we observed that the nature of this relationship appears to vary according to reproductive strategy. Notably, species that reproduce primarily asexually tended to exhibit lower levels of nucleotide diversity than would be expected given their large effective population sizes (Figure 3B). Since low diversity in these outliers cannot be explained by small *N̂*_e_, an alternative explanation would be an unusually low mutation rate in these species, relative to other eukaryotes. Many of the species that fall outside the expected trend are unicellular fungi and protists, groups that have been shown to have the lowest mutation rates among eukaryotes, generally more than an order of magnitude lower than in multicellular taxa (Wang and Obbard 2023). This may be a consequence of them having only a single cell division per generation, but could also result from more efficient selection to lower spontaneous mutation rates in large‐*N*_e_ populations, as proposed by Lynch and colleagues in the drift‐barrier hypothesis (Lynch 2010; Sung et al. 2012). Such mechanisms have previously been proposed to explain unusually low mutation rates in asexually reproducing giant duckweed (Xu et al. 2019) and brown algae (Krasovec et al. 2023). The anticipated negative relationship between *N*_e_ and mutation rates has received empirical support based on direct mutation rate data (Lynch et al. 2016, 2023; Wang and Obbard 2023), and could be a step towards explaining the patterns in our data.

### 4.3 *N*_e_ and the Efficiency of Selection

As expected, we show that a measure of the efficiency of selection, π_N_/π_S_, is correlated to *N̂*_e_. However, the relationship is perhaps not as strong as we might anticipate (*R* ^2^ = 0.26)—contrast this, for example, with the very strong correlation between π_N_/π_S_ and π_S_ across the *D. melanogaster* genome reported by Castellano et al. (2018) (note that π_N_/π_S_ and π_S_ were made statistically independent in this analysis). Several factors could contribute to the relatively weak association observed here. First, there is some evidence that the distribution of fitness effects (DFE) varies between species (Huber et al. 2017; Castellano et al. 2019). In addition, if balancing selection maintains substantial levels of genetic variation, and the prevalence of such selection varies between species, this could further weaken the relationship between π_N_/π_S_ and *N̂*_e_. Population bottlenecks and genetic hitch‐hiking may also contribute to this pattern. Both processes tend to elevate π_N_/π_S_ because deleterious genetic variation typically recovers more quickly than neutral genetic variation following reductions in diversity (Gordo and Dionisio 2005; Do et al. 2015; Brandvain and Wright 2016; Castellano et al. 2018). If the frequency or intensity of these processes varies between species, this will introduce additional noise into the relationship between π_N_/π_S_ and *N̂*_e_.

Consistent with a role for hitch‐hiking, we find that the relationship between π_N_/π_S_ and *N̂*_e_ depends on mating system, with π_N_/π_S_ being higher in asexual species relative to sexual species with comparable *N̂*_e_ (Figure 4A). The fixation of an advantageous mutation reduces genetic diversity; in sexual organisms this reduction is confined to those regions in linkage with the selected locus, whereas in asexual organisms it affects the entire genome. Following a selective sweep, genetic diversity begins to accumulate again, but as previously mentioned, deleterious variation recovers more rapidly than neutral variation (Gordo and Dionisio 2005; Do et al. 2015; Brandvain and Wright 2016; Castellano et al. 2018). As a consequence, π_N_/π_S_ is expected to be elevated for a given π_S_ and inferred *N̂*_e_ in asexual species.

There may also be alternative explanations for the elevated π_N_/π_S_ ratio observed in asexual species. In the asexual duckweed *Spirodela polyrhiza* , for example, higher π_N_/π_S_ has been attributed to relaxed selection on certain genes due to simplified morphology and life cycles compared to other multicellular eukaryotes, as well as a lack of competitive selection among genotypes in purely clonal populations (Ho et al. 2019). These factors may collectively reduce the efficiency of selection against weakly deleterious mutations, counteracting the expected benefits of large *N̂*_e_.

### 4.4 *N*_e_ and Genome Size

Populations with large effective population sizes are expected to experience more efficient natural selection, a pattern largely supported by our data (Figure 4A). This notion forms the basis of one of the most influential models of non‐adaptive genome size evolution (Lynch and Conery 2003; Lynch 2007). If mobile genetic elements and other genome‐expanding features can be considered to be weakly deleterious, they should have a higher probability of reaching fixation in populations with small *N*_e_ due to drift, leading to the accumulation of non‐coding DNA and, ultimately, larger genomes. This model has received mixed empirical support. Some studies have reported the expected negative correlation between genome size and *N*_e_ within specific lineages (Yi and Streelman 2005; Lefébure et al. 2017; Mérel et al. 2025). However, a growing body of work suggests that *N*_e_ may not be a consistent determinant of genome size, based on studies using indirect measures of *N*_e_ including *N*_e_μ, census population size and dN/dS (Whitney et al. 2010; Whitney and Garland 2010; Roddy et al. 2021; Marino et al. 2024).

Here we have added to this literature using mutation‐corrected *N*_e_ estimates, which overcome the potential confounding influence of interspecific variation in the mutation rate. After accounting for phylogenetic non‐independence using PGLS, we found no significant relationship between *N̂*_e_ and genome size (Figure 4B). This finding is consistent with that of Whitney and Garland (2010), who demonstrated that the original significant negative relationship reported by Lynch and Conery (2003) was absent when the data were reanalysed using PGLS. This implies that a strong phylogenetic signal in genome sizes may be inflating the apparent strength of the relationship between the two traits in the absence of phylogenetic correction.

However, it is also possible that the timescale over which our *N̂*_e_ estimates operate is not appropriate for detecting the true relationship with genome size, as noted by Marino et al. (2024). Processes such as transposable element accumulation, segmental duplications and other genomic expansions occur gradually over millions of years of evolution. In contrast, ‘long‐term’ *N*_e_ estimates based on nucleotide diversity reflect population sizes over timescales on the order of *4N*_e_ generations—the age of the current diversity. This may be far shorter than the evolutionary span over which genome size evolves. Furthermore, the fact that we detect no phylogenetic signal in *N̂*_e_ within lineages (Table 1) suggests that *N̂*_e_ can change relatively rapidly across phylogenetic space and may be influenced by recent demographic events. Such fluctuations may also obscure any true long‐term relationship with genome size, if such a relationship does exist.

### 4.5 Short‐ and Long‐Term Estimates of *N*_e_

Effective population sizes can be estimated over different timescales (Nadachowska‐Brzyska et al. 2022). Contemporary *N*_e_, often estimated from changes in allele frequency over time or patterns of linkage disequilibrium (Hill 1981; Waples 1989), represents *N*_e_ over the previous few generations. These estimates are typically in the hundreds of individuals or even fewer (Palstra and Ruzzante 2008; Clarke et al. 2024), which is substantially lower than our long‐term *N̂*_e_ estimates. We find no correlation between our estimates and contemporary equivalents (*n* = 17, *R* ^2^ = 0.17, *p* = 0.1, Figure S5). There are several possible explanations for this discrepancy. As Waples (2024) notes, contemporary methods are often downwardly biassed because when true *N*_e_ is large, these methods frequently produce infinite estimates, which tend to go unreported in the literature. However, this reporting bias is unlikely to account for the full extent of the mismatch. A more fundamental explanation may lie in subtle population structure. Gene flow is often spatially restricted; individuals and gametes typically disperse over relatively short distances (Endler 1977). As a result, when contemporary *N*_e_ is estimated by sampling over a few generations in one locality, only the fluctuations in allele frequencies in that part of the species' range will be measured. Over short timescales this substructure should reduce the apparent *N*_e_. Over longer timeframes, however, even modest levels of gene flow through migration can homogenise allele frequencies across subpopulations (Waples 2010), resulting in an effectively panmictic population and a much larger estimate of long‐term *N*_e_.

This raises the question of which *N*_e_ is more useful. Genetic drift is a key force both in the context of long‐term evolutionary dynamics and in conservation genetics. By definition, long‐term *N*_e_ gives us more information about the evolutionary processes that have shaped current levels of neutral diversity, since that is the data from which it is calculated. In contrast, short‐term *N*_e_ is informative about processes that equilibrate on a faster timescale, such as levels of linkage disequilibrium. This is also the effective size that is relevant for assessing extinction risk in contemporary populations, as it reflects the levels of genetic drift and inbreeding depression that are expected in the near future. This has led to the incorporation of contemporary *N*_e_ data into population viability assessments and conservation management practices through frameworks such as the 50/500 rule (Franklin 1980; Hoban et al. 2020; Convention on Biological Diversity 2022; Clarke et al. 2024).

Finally, the distinction between these timescales is critical when considering the efficacy of natural selection, as we have done in this study. While larger *N*_e_ theoretically leads to more effective selection, the spatial scale over which selection operates is less clear. Local adaptation is evidently widespread, and in such cases the ‘local’ *N*_e_, which is captured by contemporary estimates, may be more relevant in determining the immediate outcome of selective episodes. However, the large phenotypic differences observed between species and the presence of many adaptive amino acid substitutions fixed across species ranges (Galtier 2016; Rousselle et al. 2020) indicate that some level of selection continues to act on a more global basis. This is supported by the significant relationship we identified between π_N_/π_S_ and our species‐wide, long‐term *N̂*_e_ estimates (Figure 4A). Ultimately, both contemporary and long‐term *N*_e_ estimates are useful but should be carefully interpreted in light of the temporal and spatial scales that they reflect.

## Author Contributions

A.E.‐W. conceptualised the project, L.L. performed the data curation and formal analysis, L.L. wrote the first draft with input from A.E.‐W., both authors edited the manuscript, A.E.‐W. supervised the research.

## Funding

The authors have nothing to report.

## Conflicts of Interest

The authors declare no conflicts of interest.

## Supporting information


**Figure S1:** Correlation matrix for the demographic and life history traits included in main Table 2.
**Figure S2:** Plotting nucleotide diversity (π) against estimated effective population size (*N̂*e) without the issue of correlated sampling errors. (A) and (B) use separate nucleotide diversity datasets for the y‐axis (plotted directly) and the *x*‐axis (used to calculate *N̂*e), that is, Nucleotide diversity #1 was used to calculate Effective population size #1, while Nucleotide diversity #2 was used to calculate Effective population size #2.
**Figure S3:** PGLS regression of the ratio non‐synonymous to synonymous diversity (π_N_/π_S_) on estimated effective population size (*N̂*e) on a log–log scale. Species are coloured based on their reproductive strategy.
**Figure S4:** Key analyses of the paper reperformed on a subset of the species for which there is limited population structure in the sampling of the nucleotide diversity estimates (*n* = 46). (A) Histogram of *N̂*e estimates across all species plotted on a logarithmic scale. (B) PGLS regression of nucleotide diversity (π) on estimated effective population size (*N̂*e) on a log–log scale. Species are plotted based on their reproductive strategy. (C) PGLS regressions of estimated effective population size (*N̂*e) on three key traits: generation time (left), propagule size (middle) and species range divided by body mass (right).
**Figure S5:** Testing for a correlation between contemporary and long‐term estimates of effective population size (*N̂*e). Contemporary estimates for each species are the median estimate from Clarke et al. (2024), long‐term estimates can be found in Table 1.

**Table S1:** Ne estimates w/confidence intervals. Calculated using nucleotide diversity (mean_pi) and genome‐wide mutation rates (u_mean).
**Table S2:** Direct mutation rate estimates sourced from the literature.
**Table S3:** All nucleotide diversity estimates sourced from the literature.
**Table S4:** Data on life history traits and genome size sourced from the literature.
**Table S5:** Population density and species range data sourced from the literature or calculated from the data in Table S6.
**Table S6:** Range size estimates produced using GBIF occurrence records and the R package gbif.range.
**Table S7:** Results from phylogenetically‐controlled multiple regression analyses looking at how pairs of traits can explain variation in *N̂*e.

## Data Availability

The data and R code required to replicate the analysis in this article are available on GitHub (https://github.com/lovedayel/comparative‐Ne).
