# Supplementary material for: A Comparative Analysis of Long‐Term Effective Population Sizes Across Eukaryotes
Source: Mol Ecol. 2026 Feb 12;35(4):e70265. doi: 10.1111/mec.70265 (PMC12895298; doi:10.1111/mec.70265)
Supplement: Supplementary file 1 — Figure S1: Correlation matrix for the demographic and life history traits included in main Table 2. Figure S2: Plotting nucleotide diversity (π) against estimated effective population size (N̂e) without the issue of correlated sampling errors. (A) and (B) use separate nucleotide diversity datasets for the y‐axis (plotted directly) and the x‐axis (used to calculate N̂e), that is, Nucleotide diversity #1 was used to calculate Effective population size #1, while Nucleotide diversity #2 was used to calculate Effective population size #2. Figure S3: PGLS regression of the ratio non‐synonymous to synonymous diversity (πN/πS) on estimated effective population size (N̂e) on a log–log scale. Species are coloured based on their reproductive strategy. Figure S4: Key analyses of the paper reperformed on a subset of the species for which there is limited population structure in the sampling of the nucleotide diversity estimates (n = 46). (A) Histogram of N̂e estimates across all species plotted on a logarithmic scale. (B) PGLS regression of nucleotide diversity (π) on estimated effective population size (N̂e) on a log–log scale. Species are plotted based on their reproductive strategy. (C) PGLS regressions of estimated effective population size (N̂e) on three key traits: generation time (left), propagule size (middle) and species range divided by body mass (right). Figure S5: Testing for a correlation between contemporary and long‐term estimates of effective population size (N̂e). Contemporary estimates for each species are the median estimate from Clarke et al. (2024), long‐term estimates can be found in Table 1. [file MEC-35-e70265-s002.docx]

**Supplementary figures**

**A comparative analysis of long-term effective population sizes across eukaryotes**

Authors: Loveday Lewin & Adam Eyre-Walker

**Figures S1 – S5**

**Supplementary Figure 1**. Correlation matrix for the demographic and life history traits included in main Table 2.

**Supplementary Figure 2.** Plotting nucleotide diversity (π) against estimated effective population size (*N̂_e_*) without the issue of correlated sampling errors. [A] and [B] use separate nucleotide diversity datasets for the y-axis (plotted directly) and the x-axis (used to calculate *N̂_e_*), i.e. Nucleotide diversity #1 was used to calculate Effective population size #1, while Nucleotide diversity #2 was used to calculate Effective population size #2.

**Supplementary Figure 3.** PGLS regression of the ratio non-synonymous to synonymous diversity (π_N_/π_S_) on estimated effective population size (*N̂_e_*) on a log-log scale. Species are coloured based on their reproductive strategy.

**Supplementary Figure 4.** Key analyses of the paper reperformed on a subset of the species for which there is limited population structure in the sampling of the nucleotide diversity estimates (n = 46). [A] Histogram of *N̂_e_* estimates across all species plotted on a logarithmic scale. [B] PGLS regression of nucleotide diversity (π) on estimated effective population size (*N̂_e_*) on a log-log scale. Species are plotted based on their reproductive strategy. [C] PGLS regressions of estimated effective population size (*N̂_e_*) on three key traits: generation time (left), propagule size (middle) and species range divided by body mass (right).

**Supplementary Figure 5.** Testing for a correlation between contemporary and long-term estimates of effective population size (*N̂_e_*). Contemporary estimates for each species are the median estimate from Clarke et al. (2024), long-term estimates can be found in Supplementary Table 1.

**Reference:**

Clarke SH et al. 2024. Global assessment of effective population sizes: Consistent taxonomic differences in meeting the 50/500 rule. Mol. Ecol. 33:e17353. doi: 10.1111/mec.17353.
